# Magnetic resonance imaging after ligation of the intersphincteric fistula tract for high perianal fistulas in Crohn's disease: a retrospective cohort study

**DOI:** 10.1111/codi.15296

**Published:** 2020-08-29

**Authors:** E. M. Meima ‐ van Praag, K. L. van Rijn, M. A. Monraats, C. J. Buskens, J. Stoker

**Affiliations:** ^1^ Department of Surgery Amsterdam Gastroenterology, Endocrinology and Metabolism Amsterdam UMC University of Amsterdam Amsterdam The Netherlands; ^2^ Department of Radiology and Nuclear Medicine Amsterdam Gastroenterology, Endocrinology and Metabolism Amsterdam UMC University of Amsterdam Amsterdam The Netherlands

**Keywords:** Perianal fistula, ligation of intersphincteric tract, magnetic resonance imaging, perianal Crohn’s disease

## Abstract

**Aim:**

Ligation of the intersphincteric fistula tract (LIFT) is increasingly being used for surgical closure of high perianal fistulas in Crohn’s disease. Currently, data on postoperative MRI findings are scarce, although they are considered important for assessing healing and recurrence. Our aim, therefore, was to evaluate fistula characteristics on MRI and their relationship with clinical outcomes after LIFT.

**Method:**

Consecutive Crohn's patients treated with LIFT between 2007 and 2018 who underwent baseline and follow‐up MRI were retrospectively included. MRIs were scored by two radiologists according to characteristics based on the original and modified Van Assche indices. MRI findings, with emphasis on fibrosis, and the relationship with clinical healing, re‐interventions and recurrences are described.

**Results:**

Twelve patients were included [four men, median age 34 (interquartile range 28–39) years]. Follow‐up MRI was performed at a median of 5.5 months (interquartile range 2.5–6.0) after LIFT. At baseline, all patients showed a tract with predominantly granulation tissue, which changed to predominantly fibrotic in seven (in three of whom it was completely fibrotic). All patients with a (predominantly) fibrotic tract had clinical closure and no re‐interventions or recurrences during long‐term follow‐up. In contrast, of the five patients with persisting granulation tissue, two reached clinical healing, two needed re‐intervention and one had a recurrence.

**Conclusion:**

Markedly decreased fistula activity can be observed on MRI after LIFT. The majority of patients develop a predominantly fibrotic tract relatively soon after LIFT without clinical recurrence, suggesting a highly effective therapy. Unfavourable clinical outcomes were only present in patients with persisting granulation tissue, indicating the potential prognostic value of MRI.

**What does this paper add to the literature?**

There is no current consensus on the interpretation of MRI after ligation of the intersphincteric fistula tract. This paper provides an understanding of fistula behaviour on MRI. Most patients show predominantly fibrotic tracts after a median of 5.5 months and have an absence of recurrence, suggesting effective therapy. Persisting granulation tissue was associated with worse clinical outcomes, indicating the prognostic value of MRI.

## Introduction

Crohn's perianal fistulas are a continuing challenge for treating physicians, with a total incidence of around 25% in Crohn's patients and even higher in patients with colonic or rectal involvement [1, 2]. Sphincter‐preserving surgery is increasingly used to treat high perianal fistulas, preceded by several weeks of drainage using a noncutting seton and optimization of medical therapy. So far, most reports describe results with an endorectal advancement flap (AF) [3, 4, 5], but the more novel technique of ligation of the intersphincteric fistula tract (LIFT) is a promising alternative sphincter‐preserving procedure. The LIFT procedure, during which the fistula tract is identified and ligated in the intersphincteric plane [6], is increasingly used in clinical practice. Unfortunately, there is limited information on clinical and radiological follow‐up after LIFT.

In clinical practice, MRI is the preferred diagnostic modality for assessing perianal fistulas in patients with Crohn's disease due to its accuracy, noninvasiveness and ability to evaluate the entire pelvic region, as stated by global as well as European guidelines [7, 8, 9]. So far, there are limited data on the behaviour and structural assessment of perianal fistulas after the LIFT procedure. The clinical and radiological healing, recurrence and incontinence rates of Crohn's patients undergoing LIFT and AF were evaluated in a retrospective study [10]. This study demonstrated MRI healing in 53% of LIFT procedures; however, no standardized assessment of radiological healing was used. Another recent trial looking at clinical and radiological healing in a group treated with the surgeon's preferred technique showed healing on MRI in 42% of patients 12 months after LIFT [11]. Unfortunately, the definition of healing on MRI was not specified in that study.

There are several systematic approaches available for assessing the response to therapy on MRI: the Van Assche score, the modified Van Assche score and the recently developed MAGNIFI‐CD score [12, 13, 14]. The first two were developed to evaluate anti‐inflammatory therapy [13, 14] and the third in a cohort receiving stem cell therapy [12]. These scoring systems have several overlapping items; however, none of these systems focuses on surgical follow‐up, even though MRI findings can be different from other treatments due to preexisting surgical indications (for LIFT the presence of a high, usually transsphincteric, fistula tract) and insurmountable postoperative changes shortly after surgery that resemble inflammation. Therefore, scoring systems in their current form may not be applicable for patients undergoing sphincter‐preserving surgery.

MRI is considered the most important method for the assessment of healing and recurrences, but there is limited information on fistula characteristics on MRI and the relationship with clinical outcomes after LIFT surgery. Furthermore, no consensus on systematic postoperative review of MRI scans exists, while knowledge of fistula characteristics can help to guide postoperative treatment strategy. Therefore, there is a need to investigate the behaviour of fistulas on MRI for use in systematic evaluation and treatment decisions in patients after a LIFT procedure.

We aimed to assess fistula behaviour after LIFT on MRI by using structured radiological assessment, and evaluate the trend towards clinical healing and recurrence in patients with Crohn's high perianal fistulas.

## Method

### Study design and population

We performed a sub‐study of a previously performed retrospective cohort study conducted at the Amsterdam University Medical Centres [10]. That study included all consecutive Crohn's patients treated for high perianal fistulas with LIFT or AF between January 2007 and February 2018. Exclusion criteria were the presence of a cryptoglandular fistula, low perianal fistula, ileo‐anal pouch fistula, rectovaginal or recto‐urinary fistula and patients with proctitis or human immunodeficiency virus. In the current study, patients who underwent a LIFT procedure in the previously performed retrospective cohort study were evaluated and patients with AF were excluded, since a homogeneous group was desirable for the evaluation of postoperative effects on MRI. Furthermore, patients were excluded from analysis when the MRI examination was insufficient: a pre‐ or postoperative MRI was missing, important sequences were missing (such as a postcontrast T1‐weighted sequence) or the interval between the surgery and postoperative MRI was > 12 months. No formal ethical approval was needed for this study according to the Medical Ethical Committee at the Amsterdam UMC, location Academic Medical Centre. All eligible patients were contacted prior to the original retrospective study and were asked to withhold permission for data collection if they objected. Patient demographics, details of medical therapy and follow‐up were obtained from medical notes. Outcome variables included clinical healing, re‐interventions and recurrences.

### MRI assessment

#### Protocol

The clinical MRI protocol in our centre consists of sagittal T2‐weighted, coronal T2‐weighted and axial T2‐weighted sequences, an axial T2‐weighted sequence with fat suppression and a T1‐weighted postcontrast sequence. The coronal and axial sequences were angulated corresponding to the anatomy of the axis of the anal canal. MRI scans were performed on a 1.5 T scanner (Magnetom Avanto, Siemens, Erlangen, Germany). The MRI protocol remained unchanged during the study period.

#### Scoring

We a priori made up a scoring system (Table 1) from existing items of the original and modified Van Assche scores with the addition of new items [13, 14]. The MAGNIFI‐CD score was not available at the time of scoring and these items could therefore not be included [12]. First, the items that were deemed inapplicable for assessment after LIFT surgery were excluded. These items were ‘location’, ‘extension’, ‘rectal wall involvement’ and ‘presence of a recto/anovaginal tract’, because the first two were determined by the indication for a LIFT procedure (high transsphincteric tract, i.e. positioned in the upper two‐thirds of the external sphincter or puborectalis muscle) and the last two are contraindications for undergoing LIFT (i.e. proctitis and a rectovaginal tract).

**Table 1:** Scoring items.

Item	Options
Number of fistula tracts	None
	Single, unbranched
	Single, branched
	Multiple
Hyperintensity T2	Absent
	Mild
	Pronounced
Changes in volume and/or hyperintensity on T2*	Substantial decrease
	Minor decrease
	Comparable
	Minor increase
	Substantial increase
Inflammatory mass	Absent
	Diffuse
	Focal
	Collection small
	Collection medium
	Collection large
Changes in inflammatory mass*	No mass
	Same mass
	New mass (with location)
Hyperintensity on T1	Absent
	Mild
	Pronounced
Changes in volume and/or hyperintensity on postcontrast T1*	Substantial decrease
	Minor decrease
	Comparable
	Minor increase
	Substantial increase
Dominant feature	Fibrous
	Granulation tissue
	Fluid/pus

* These items were only scored on the follow‐up MRI.

Subsequently, several examples not included in this study were reviewed by two experienced abdominal radiologists (JS and MM) in consensus to observe if these items were suitable for capturing changes in activity between baseline and follow‐up MRI. In this session, three new scoring items were defined to capture postoperative changes: two items measuring changes in activity and volume on T2‐weighted and postcontrast T1‐weighted sequences on a five‐point scale and one item capturing changes in inflammatory masses (indicating if there was a new mass and, if present, where it was located). Diagnostic scan quality was assessed as ‘good’, ‘adequate’ or ‘poor’ for all scans. Good quality was defined as optimal diagnostic quality without artefacts (e.g. from patient movement in the scanner), adequate quality was defined as sufficient diagnostic quality but with some artefacts and poor quality was defined as insufficient diagnostic quality to assess the fistula.

Hereafter, all scans (baseline and follow‐up) were scored in a random order by two abdominal radiologists (JS and MM) in consensus. Radiologists were blinded to the results of clinical follow‐up. Subsequently, the items measuring transformations between baseline and follow‐up were addressed per patient, comparing the baseline and follow‐up scan. As this concerned the first step in developing a LIFT‐dedicated MRI score, no assessment of reproducibility was performed.

### Clinical assessment

Clinical healing was defined as closure of the external opening without discharge of pus or faeces, as assessed from the medical notes of the treating surgeon during postoperative appointments by a research fellow (EMMP). The date on which clinical healing was first described in the medical notes was considered the clinical healing date. Recurrence was defined as reopening of the external fistula or recurrence of symptoms after apparent healing, and re‐intervention was defined as any surgical redo procedure for fistula‐related complaints. Healing and recurrences were assessed during postoperative follow‐up, consisting of scheduled outpatient appointments at 6 weeks and 3 and 6 months, or when patients contacted their surgeon with (reoccurrence or worsening of) symptoms.

### Statistical analysis

All data were collected in an electronic database. The clinical and MRI findings are described and are presented as frequencies with percentages and medians with interquartile ranges (IQR). The relationship of MRI with clinical parameters is described. Analyses were performed using IBM® spss® for Windows® version 24 (IBM Corp., Armonk, New York, USA).

## Results

### Baseline characteristics

Out of 19 patients who underwent a LIFT procedure for Crohn's high perianal fistulas between 2007 and February 2018, 12 were included in this study. Four patients were excluded due to missing pre‐ or postoperative MRI examinations and three due to missing sequences (MRIs performed elsewhere). Patient characteristics are shown in Table 2.

**Table 2:** Demographics (for the LIFT procedure) (*n* = 12).

Male sex, *n* (%)	4 (33)
Age, years*	34 (28–39)
History of smoking, *n* (%)	6 (50)
Smoking at moment of surgery^†^, *n* (%)	5 (42)
Familial inflammatory bowel disease, *n* (%)	3 (25)
Stoma, *n* (%)	3 (25)
Location of Crohn's disease, *n* (%)	
L1 ileum	3 (25)
L2 colon	1 (8)
L3 both	3 (25)
Missing	5 (42)
Use of anti‐TNF, *n* (%)	10 (83)
Use of immunomodulators, *n* (%)	7 (58)
Preoperative seton drainage, months*	6 (3–13)

LIFT, Ligation of the intersphincteric fistula tract; TNF, tumour necrosis factor.

* Values are median (interquartile range).

^†^ Or within 3 months prior to surgery.

All patients underwent preoperative seton drainage for a median of 6.0 months (IQR 3.4–13.3 months) and all but one used immunomodulators and/or anti‐tumour necrosis factor (TNF) at the time of surgery. From these 11 patients on medication during their surgery, five stopped their immunomodulatory and/or anti‐TNF within 3 months postoperatively. No complications occurred during surgery or within 90 days after surgery.

### MRI assessment

The interval between LIFT surgery and the (first) follow‐up MRI was a median of 5.5 months (IQR 2.5–6.0 months). The reasons for performing the first postoperative MRI were to assess radiological healing in 11 patients and to assess if there was a recurrence in the remaining patient (Table 3, patient 4). Two patients had two follow‐up MRIs, both after 2 months, and a second follow‐up MRI at 8 and 18 months, respectively. In one patient, the second MRI was performed to assess closure and the possibility of stoma reversal. In the other patient, the second follow‐up MRI was performed according to a protocol related to another study [15].

**Table 3:** Baseline and follow‐up MRI scores per patient.

Patient	Baseline MRI				Follow‐up MRI(s)							Clinical healing
	Predominant feature	Hyperintensity T2	Hyperintensity T1	Inflammatory mass	Time since surgery (months)	Predominant feature	Hyperintensity T2	Changes volume/ hyperintensity T2	Hyperintensity T1	Changes volume/hyperintensity T1	Inflammatory mass	
1	Granulation tissue	Pronounced	Pronounced	Absent	11	Fibrous	Absent	Substantial decrease	Absent	Substantial decrease	Absent	Yes
2	Granulation tissue	Pronounced	Pronounced	Absent	6	Fibrous	Absent	Substantial decrease	Absent	Substantial decrease	Absent	Yes
3	Granulation tissue	Mild	Mild	Focal	5	Fibrous	Absent	Substantial decrease	Absent	Substantial decrease	Absent	Yes
4	Granulation tissue	Pronounced	Pronounced	Absent	2	Fibrous	Mild	Substantial decrease	Mild	Minor decrease	Absent	Yes
					18	Fibrous	Absent	Substantial decrease	Absent	Substantial decrease	Absent	
5	Granulation tissue	Mild	Mild	Absent	6	Fibrous	Mild	Minor decrease	Mild	Minor decrease	Absent	Yes
6	Granulation tissue	Pronounced	Pronounced	Focal	5	Fibrous	Pronounced	Minor decrease	Pronounced	Minor decrease	Absent	Yes
7	Granulation tissue	Pronounced	Pronounced	Focal	2	Fibrous	Pronounced	Minor decrease	Pronounced	Minor decrease	Focal (same) and small collection (new, intersphincteric)	Yes
					8	Fibrous	Mild	Substantial decrease	Mild	Substantial decrease	Focal (same)	
8	Granulation tissue	Pronounced	Pronounced	Diffuse	6	Granulation tissue	Pronounced	Minor decrease	Pronounced	Comparable	Absent	Yes (later recurrence)
9	Granulation tissue	Mild	Mild	Focal	2	Granulation tissue	Mild	Minor decrease	Mild	Minor decrease	Focal (same)	No (later yes)
10	Granulation tissue	Mild	Mild	Absent	8	Granulation tissue	Mild	Comparable	Mild	Comparable	Absent	No (later yes)
11	Granulation tissue	Pronounced	Pronounced	Focal	6	Granulation tissue	Pronounced	Minor decrease	Pronounced	Minor decrease	Focal	No (re‐intervention)
12	Granulation tissue	Pronounced	Pronounced	Absent	4	Granulation tissue	Pronounced	Minor decrease	Pronounced	Minor decrease	Focal (new, cutaneous)	No (re‐intervention)

**Figure 1:**
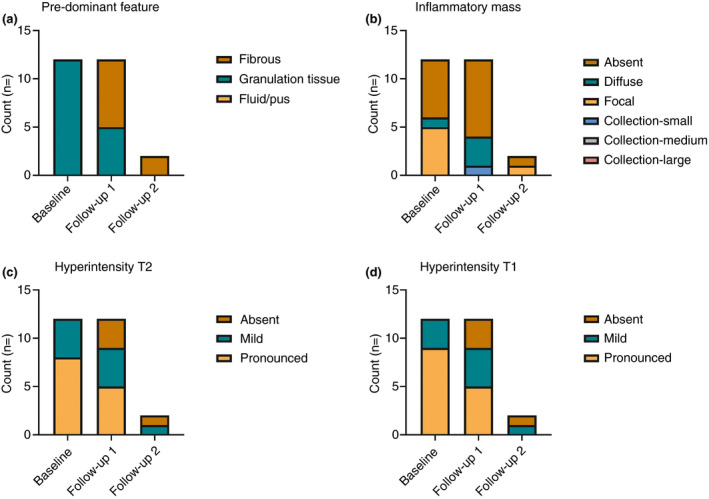
Scoring items at baseline and follow‐up MRI for all patients: (a) the predominant feature of the tract, (b) the inflammatory masses, (c) the hyperintensity scored on fat‐saturated T2‐weighted images, (d) the hyperintensity scored on fat‐saturated T1‐weighted postcontrast images.

Scan quality was good in 24/26 (92%) scans and adequate in 2/26 (8%) scans; none were classified as poor quality. Table 3 shows the MRI features per patient at baseline and follow‐up. In Fig. 1 the scores of the different items at baseline and follow‐up are displayed.

### MRI and short‐term clinical response

**Figure 2:**
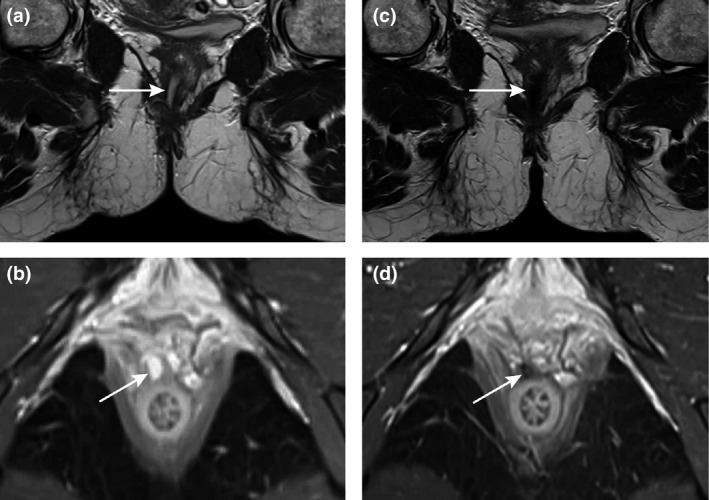
Completely fibrotic tract at 5‐months’ follow‐up. Coronal T2‐weighted images (a, c) and axial fat‐saturated T1‐weighted postcontrast images (b, d) at baseline (a, b) and at 5 months’ follow‐up (c, d) of a 34‐year‐old woman. MRI showed a fistula tract that changed from predominantly granulation tissue to predominantly fibrous 5 months after the ligation of the intersphincteric fistula tract procedure. The hyperintensity of the tract was pronounced at T1‐weighted postcontrast images at baseline (b) and absent at follow‐up (d). The patient showed clinical healing at the time of the follow‐up MRI.

**Figure 3:**
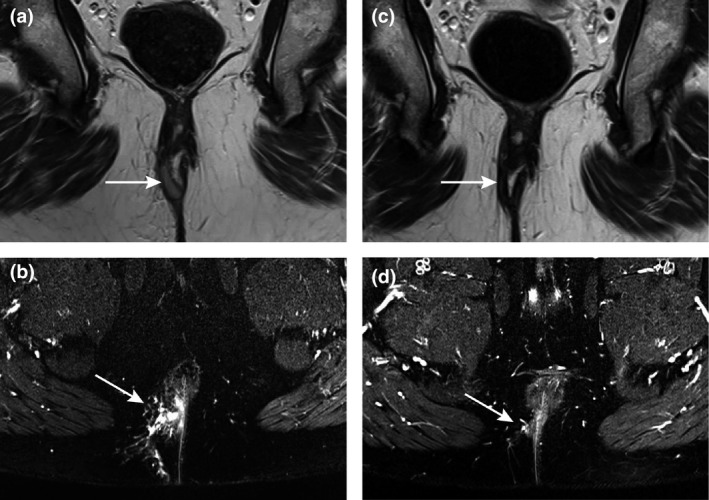
Predominantly fibrous tract at 5 months’ follow‐up. Coronal T2‐weighted images (a, c) and axial fat‐suppressed T2‐weighted images (b, d) at baseline (a, b) and at 5 months’ follow‐up (c, d) of a 37‐year‐old man. MRI showed a fistula tract that changed from predominantly granulation tissue to predominantly fibrous 5 months after the ligation of the intersphincteric fistula tract procedure, and the hyperintensity changed from pronounced to mild. Also, a focal inflammatory mass (b) was seen at baseline which disappeared at follow‐up (d). The patient showed clinical healing at the time of the follow‐up MRI.

At baseline, all patients showed a tract predominantly filled with granulation tissue. This changed to predominantly fibrotic in seven, three of whom showed a completely fibrotic tract with absence of hyperintensity on T2‐weighted and enhancement on postcontrast T1‐weighted sequences (Fig. 2). In four of these seven there was still mild or pronounced hyperintensity present at follow‐up (Fig. 3). All seven showed a decreased volume and/or hyperintensity of the tract; three a minor and four a substantial decrease on T2‐weighted imaging, and four a minor and three a substantial decrease on postcontrast T1‐weighted imaging. A focal inflammatory mass was present in three of these seven patients at baseline, which persisted in one patient. Within a year, all seven of these patients showed clinical healing after a median of 2.3 months (IQR 1.4–2.8 months) and no re‐interventions were performed.

Five patients showed persisting granulation tissue on the follow‐up MRI. Three of these patients reported clinical healing after a median follow‐up of 4.4 months (IQR 1.6–10.2 months), one of whom developed a recurrence 12 months’ postoperatively. Two patients without clinical healing required re‐interventions at 5 and 8 months’ postoperatively, due to abscess formation and persisting fistula drainage, respectively.

### Second follow‐up MRIs and long‐term clinical follow‐up

The two patients who underwent a second MRI scan had reached a predominantly fibrotic tract at the first follow‐up MRI, with pronounced and mild hyperintensity on T2‐weighted and postcontrast T1‐weighted images. At the second follow‐up MRI, one patient had a completely healed tract with an absence of hyperintensity and the other still showed mild hyperintensity. Both patients already showed clinical healing at the time of the first MRI, and there were no recurrences. One of these two patients did show a new small intersphincteric collection at the site of the LIFT surgery on the first follow‐up MRI, which was a postoperative effect. This had disappeared on the second follow‐up MRI.

Overall, after a median follow‐up of 23.7 months (16.8–43.4 months) all patients with a predominantly fibrotic tract had complete fistula closure without recurrence or re‐intervention. In contrast, in the patient group with persisting granulation tissue clinical closure was seen in only 40%.

## Discussion and conclusions

In this study, we have described the behaviour of perianal fistulas on MRI after the LIFT procedure in patients with Crohn's disease and its relationship to clinical outcomes. More than half of the patients showed a tract that transitioned from predominantly granulation tissue to predominantly fibrous after a relatively short follow‐up period (median 5.5 months). This was accompanied by clinical healing and was associated with good long‐term outcome with absence of recurrences in all these patients. Re‐interventions and recurrences all occurred in patients with postoperative persistence of granulation tissue, with only 40% healing on long‐term follow‐up, suggesting that MRI has clinical value in determining postoperative treatment strategy and, potentially, prognostic value for durable healing.

Several postoperative signs of fistula healing were shown on MRI, such as changes in hyperintensity and the predominant feature of a tract. Of this feature, a completely fibrotic tract can be considered as the most definitive sign of fistula healing on MRI. Seven out of 12 patients showed a predominantly fibrotic tract within a year after the LIFT procedure, of whom three showed a completely fibrotic tract with absence of hyperintensity on all sequences. Only one previous study evaluated healing on MRI after LIFT surgery; healing was reported for a combined group after a procedure according to surgeon preference, which was 49% after 12 months [11]. However, that involved cryptoglandular fistulas and, unfortunately, no specific MRI features and no clear definition of healing on MRI were provided, making comparison with our results difficult. We specifically looked at Crohn's fistulas in the current study because clinical and radiological follow‐up of these patients is often complicated with abscesses, recurrences and re‐interventions.

No clear consensus on fistula healing on MRI exists, and studies focusing on other treatments do not report the definition of healing or use different definitions, such as remission of activity and absence of collections > 2 cm [16]. If we look at studies evaluating MRI after anti‐TNF treatment, fibrotic tracts are reported in between 20% and 30% of patients within a year after treatment [17, 18]. This is comparable with the 25% of complete fibrotic tracts in our study, although caution should be taken when directly comparing these results due to the small sample size and selected population of our retrospective cohort.

Interestingly, we observed a predominantly, but not completely, fibrotic tract in another 30% of patients. Chambaz *et al*. [19] found that deep remission (clinical remission and absence of enhancement on MRI) is associated with higher flare‐free survival, but the value of a predominantly, but not completely, fibrotic tract on MRI is unknown. Only one retrospective study reports this feature after anti‐TNF treatment, with predominant fibrosis being seen in only 3 out of 30 patients within 18 months [20]. In our cohort, no recurrences were observed in any patients with a predominantly fibrotic tract on MRI. The high percentage (58%) of (predominant) fibrosis in our study and absence of recurrences for a median follow‐up of almost 2 years after LIFT show that this MRI feature might have a predictive value for durable healing after the LIFT procedure. These findings warrant further research in larger prospective trials evaluating LIFT surgery.

Persisting granulation tissue on follow‐up MRI was seen in five patients: two of these reached clinical healing, two needed a re‐intervention and one had a recurrence after 12 months. This suggests that the presence of persisting granulation tissue is indicative of a worse clinical outcome. This knowledge may be used in decisions on discontinuation of postoperative medical treatment or stoma reversal, and indicates that MRI can potentially play a role in formulating a treatment strategy after LIFT. However, the presence of a tract with persisting granulation tissue itself should not be an indication for re‐intervention since some of these patients still develop a fibrotic tract. It should be further evaluated whether the presence of persisting granulation tissue can indeed predict worse clinical outcome and evaluate if and when medical treatment can be stopped in a (predominantly) fibrotic tract.

We used a self‐composed list of scoring items deemed useful for surgical MRI follow‐up, based on the existing (at that time) MRI classifications developed for evaluation of anti‐inflammatory medication [13, 14]. Review of these scoring indices pointed out that several features can be considered inadequate for surgical follow‐up. For example, the presence of proctitis and of a supralevator extension are contraindications to LIFT surgery (the latter since this drastically decreases the chance of preserving anal sphincter function). Furthermore, we experienced that some of the existing activity‐based items were insensitive to capturing small changes in volume and/or hyperintensity. These small changes could possibly be relevant for the prediction of clinical outcome but this could not be shown in our small cohort since most patients showed a marked response. Also, it is important to note that in clinical practice the description of fistula anatomy is as relevant as the activity. During our study another MRI‐based system was developed, the MAGNIFI‐CD score, which might be applicable for follow‐up after surgery and should be evaluated on such a cohort [12]. Consensus on a systematic approach to review MRIs after surgical closure can help standardize any evaluation used in the assessment and prediction of clinical outcomes.

It is important to keep in mind the timing of MRI after surgical interventions during interpretation of the findings. After surgery, distinguishing normal postoperative changes and inflammatory changes on MRI can be challenging and should be carefully considered and related to the clinical condition of the patient. This is illustrated by one of the two patients who underwent a second follow‐up MRI in our study. In this patient, a new small intersphincteric collection occurred at the first MRI at the surgery site; this was interpreted as a postoperative effect and therefore had no clinical consequences and disappeared on the second follow‐up MRI. However, in some cases the identification of postoperative effects can be less clear‐cut and might be a precursor to abscess formation. This highlights the importance of keeping in mind the postoperative status when interpreting MRI results. We suggest that an MRI is performed to assess radiological healing no earlier than 3 months after the procedure, when the fistula is clinically closed and the surgeon, for example, considers stopping postoperative anti‐TNF treatment. This is with the exception of assessing for recurrences when a patient has a deterioration of fistula complaints, at which point an MRI is indicated regardless of the timing after surgery.

This study has several limitations. The cohort is small and with selected patients, which limits the ability to draw firm conclusions on the role of MRI characteristics in the postoperative treatment strategy and prediction of durable healing. Furthermore, we did not use a fully validated scoring index to assess the MRI scans, but evaluated separate items extracted from validated scoring systems and added some new items [13, 14]. Therefore, we were not able to make a meaningful comparison of changes in a total score between baseline and follow‐up. However, since existing scoring indices were not applicable to our population and it was our aim to evaluate fistula behaviour on MRI after the LIFT procedure our approach of using different items fitted our goal. However, if the MAGNIFI‐CD score had been present we would have evaluated the value of this score, noting that many of the addressed items in the current study are also part of MAGNIFI‐CD. In addition, only the short‐term behaviour of fistula tracts was assessed by MRI. In the future it would be interesting to describe fistula behaviour on MRI at longer periods of follow‐up and determine the role of MRI in the prediction of fistula healing and treatment strategy.

In conclusion, in this cohort of Crohn's patients after the LIFT procedure a clear radiological response was shown and the results demonstrate that a (predominantly) fibrotic tract was associated with good clinical outcomes, with no recurrences at long‐term follow‐up. In contrast, unfavourable clinical outcomes were only present in patients with persisting granulation tissue. These findings suggest a role for MRI in postoperative evaluation after LIFT, focusing on the extent of fibrosis, which is worth further evaluation in larger, prospective studies.

## Funding

None.

## Conflicts of interest

EMMP, KLR, MAM and CJB have nothing to declare. JS has a research agreement with Takeda not related to this topic.

## Supporting information


**Appendix S1**. Definitions of scoring items.

## Data Availability

The data that support the findings of this study are available in the article and the supplementary material of this article (Appendix S1).

## References

[1] Hellers G , Bergstrand O , Ewerth S , Holmstrom B . Occurrence and outcome after primary treatment of anal fistulae in Crohn's disease. Gut 1980; 21: 525–7. 7429313 10.1136/gut.21.6.525 PMC1419665

[2] Schwartz DA , Loftus EV Jr , Tremaine WJ *et al* The natural history of fistulizing Crohn's disease in Olmsted County, Minnesota. Gastroenterology 2002; 122: 875–80. 11910338 10.1053/gast.2002.32362

[3] Bemelman WA , Warusavitarne J , Sampietro GM *et al* ECCO‐ESCP consensus on surgery for Crohn's disease. J Crohn Colitis 2018; 12: 1–16. 10.1093/ecco-jcc/jjx061 28498901

[4] Christoforidis D , Pieh MC , Madoff RD , Mellgren AF . Treatment of transsphincteric anal fistulas by endorectal advancement flap or collagen fistula plug: a comparative study. Dis Colon Rectum 2009; 52: 18–22. 19273951 10.1007/DCR.0b013e31819756ac

[5] Stellingwerf ME , van Praag EM , Tozer PJ , Bemelman WA , Buskens CJ . Systematic review and meta‐analysis of endorectal advancement flap and ligation of the intersphincteric fistula tract for cryptoglandular and Crohn's high perianal fistulas. BJS Open 2019; 3: 231–41. 31183438 10.1002/bjs5.50129 PMC6551488

[6] van Praag EM , Buskens CJ . The LIFT procedure for a perianal Crohn's fistula ‐ a video vignette. Colorectal Dis 2019; 21: 853–4. 31055867 10.1111/codi.14660

[7] Gecse KB , Bemelman W , Kamm MA *et al* A global consensus on the classification, diagnosis and multidisciplinary treatment of perianal fistulising Crohn's disease. Gut 2014; 63: 1381–92. 24951257 10.1136/gutjnl-2013-306709

[8] Maaser C , Sturm A , Vavricka SR *et al* ECCO‐ESGAR guideline for diagnostic assessment in IBD Part 1: initial diagnosis, monitoring of known IBD, detection of complications. J Crohn Colitis 2019; 13: 144–64. 10.1093/ecco-jcc/jjy113 30137275

[9] Kim DH , Carucci LR , Baker ME *et al* ACR appropriateness criteria Crohn disease. J Am Coll Radiol 2015; 12: 1048–57.e4. 26435118 10.1016/j.jacr.2015.07.005

[10] van Praag EM , Stellingwerf ME , van der Bilt JDW , Bemelman WA , Gecse KB , Buskens CJ . Ligation of the intersphincteric fistula tract and endorectal advancement flap for high perianal fistulas in Crohn's disease ‐ A retrospective cohort study. J Crohn Colitis 2020; 14: 757–63. 10.1093/ecco-jcc/jjz181 PMC7346888 31696918

[11] Jayne DG , Scholefield J , Tolan D *et al* Anal fistula plug versus surgeon's preference for surgery for trans‐sphincteric anal fistula: the FIAT RCT. Health Technol Assess 2019; 23: 1–76. 10.3310/hta23210 PMC6545498 31113531

[12] Hindryckx P , Jairath V , Zou G *et al* Development and validation a magnetic resonance index for assessing fistulas in patients with Crohn's disease. Gastroenterology 2019; 157: 1233–44. 31336124 10.1053/j.gastro.2019.07.027

[13] Van Assche G , Vanbeckevoort D , Bielen D *et al* Magnetic resonance imaging of the effects of infliximab on perianal fistulizing Crohn's disease. Am J Gastroenterol 2003; 98: 332–9. 12591051 10.1111/j.1572-0241.2003.07241.x

[14] Samaan MA , Puylaert CAJ , Levesque BG *et al* The development of a magnetic resonance imaging index for fistulising Crohn's disease. Aliment Pharmacol Ther 2017; 46: 516–28. 28653753 10.1111/apt.14190

[15] de Groof EJ , Buskens CJ , Ponsioen CY *et al* Multimodal treatment of perianal fistulas in Crohn's disease: seton versus anti‐TNF versus advancement plasty (PISA): study protocol for a randomized controlled trial. Trials 2015; 16: 366. 26289163 10.1186/s13063-015-0831-x PMC4545975

[16] Panes J , Garcia‐Olmo D , Van Assche G *et al* Long‐term efficacy and safety of stem cell therapy (Cx601) for complex perianal fistulas in patients with Crohn's disease. Gastroenterology 2018; 154: 1334–42 e4. 29277560 10.1053/j.gastro.2017.12.020

[17] Tozer P , Ng SC , Siddiqui MR *et al* Long‐term MRI‐guided combined anti‐TNF‐alpha and thiopurine therapy for Crohn's perianal fistulas. Inflamm Bowel Dis 2012; 18: 1825–34. 22223472 10.1002/ibd.21940

[18] Ng SC , Plamondon S , Gupta A *et al* Prospective evaluation of anti‐tumor necrosis factor therapy guided by magnetic resonance imaging for Crohn's perineal fistulas. Am J Gastroenterol 2009; 104: 2973–86. 19755971 10.1038/ajg.2009.509

[19] Chambaz M , Verdalle‐Cazes M , Desprez C *et al* Deep remission on magnetic resonance imaging impacts outcomes of perianal fistulizing Crohn's disease. Dig Liver Dis 2019; 51: 358–63. 30612820 10.1016/j.dld.2018.12.010

[20] van Rijn KL , Lansdorp CA , Tielbeek JAW *et al* Evaluation of the modified Van Assche index for assessing response to anti‐TNF therapy with MRI in perianal fistulizing Crohn's disease. Clin Imaging 2020; 59: 179–87. 31821976 10.1016/j.clinimag.2019.10.007

