# Supplementary material for: Magnetic resonance imaging after ligation of the intersphincteric fistula tract for high perianal fistulas in Crohn's disease: a retrospective cohort study
Source: Colorectal Dis. 2020 Aug 29;23(1):169–77. doi: 10.1111/codi.15296 (PMC7891352; doi:10.1111/codi.15296)
Supplement: Supplementary file 1 — Appendix S1. Definitions of scoring items. [file CODI-23-169-s001.docx]

**Appendix S1 - Definitions of scoring items**

| Item | Options | Definition |
| --- | --- | --- |
| Number of fistula tracts | None  Single, unbranched  Single, branched  Multiple |  |
| Hyperintensity T2 | Absent  Mild  Pronounced | No hyperintensity visible, only scar tissue  Slight increase in signal intensity but less than nearby, in-plane vessels  Tract showing equal or greater signal hyperintensity than nearby in-plane vessels |
| Changes in volume and/or hyperintensity on T2* | Substantial decrease  Minor decrease  Comparable  Minor increase  Substantial increase | Obvious decrease in volume and/or hyperintensity  Subtle decrease in volume and/or hyperintensity  Comparable volume and/or hyperintensity  Subtle increase in volume and/or hyperintensity  Obvious increase in volume and/or hyperintensity |
| Inflammatory mass | Absent  Diffuse  Focal  Collection small  Collection medium  Collection large | No inflammatory mass  Diffuse inflammation of surrounding tissues  Lesion >3 mm in diameter on T2-weighted images (but does not include linear tracts with diameter >3 mm) with diffuse enhancement on T1-weighted post-contrast images (ie, granulation tissue)  Circumscribed cavity 3-10 mm in diameter (but does not include linear tracts with diameter >3 mm and if present they should be excluded from the measurement of the size of the infiltrated). Hyperintense appearance on fat-saturated T2-weighted images  with enhancement limited to the rim on T1-weighted post-contrast images  As defined above except diameter measures 11-20 mm  As defined above except diameter measures >20 mm |
| Changes in inflammatory mass* | No mass  Same mass  New mass (with location) |  |
| Hyperintensity on T1 | Absent  Mild  Pronounced | No hyperintensity visible, only scar tissue  Slight increase in signal intensity but less than nearby, in-plane vessels  Tract showing equal or greater signal hyperintensity than nearby in-plane vessels |
| Changes in volume and/or hyperintensity on post-contrast T1* | Substantial decrease  Minor decrease  Comparable  Minor increase  Substantial increase | Obvious decrease in volume and/or hyperintensity  Subtle decrease in volume and/or hyperintensity  Comparable volume and/or hyperintensity  Subtle increase in volume and/or hyperintensity  Obvious increase in volume and/or hyperintensity |
| Dominant feature | Fibrous  Granulation tissue  Fluid/pus | > 50% of tract has a fibrotic appearance (ie, hypointense on fat-saturated T2-weighted images)  > 50% of tract is filled with granulation tissue (ie, hyperintense on fat-saturated T2-weighted images with enhancement of contents and wall on T1-weighted post-contrast images)  > 50% of tract is filled with fluid or pus (ie, hyperintense on fat-saturated T2-weighted images with no enhancement of contents on fat-saturated T1-weighted post-contrast images [though lining of tract may enhance]) |
| * These items were only scored on the follow-up MRI | | |
